# Matching Pursuit Analysis of Auditory Receptive Fields' Spectro-Temporal Properties

**DOI:** 10.3389/fnsys.2017.00004

**Published:** 2017-02-09

**Authors:** Jörg-Hendrik Bach, Birger Kollmeier, Jörn Anemüller

**Affiliations:** ^1^Medizinische Physik, Universität OldenburgOldenburg, Germany; ^2^Cluster of Excellence Hearing4all, Universität OldenburgOldenburg, Germany

**Keywords:** auditory receptive fields, spectro-temporal patterns, Gabor filters, matching pursuit, acoustic event classification

## Abstract

Gabor filters have long been proposed as models for spectro-temporal receptive fields (STRFs), with their specific spectral and temporal rate of modulation qualitatively replicating characteristics of STRF filters estimated from responses to auditory stimuli in physiological data. The present study builds on the Gabor-STRF model by proposing a methodology to quantitatively decompose STRFs into a set of optimally matched Gabor filters through matching pursuit, and by quantitatively evaluating spectral and temporal characteristics of STRFs in terms of the derived optimal Gabor-parameters. To summarize a neuron's spectro-temporal characteristics, we introduce a measure for the “diagonality,” i.e., the extent to which an STRF exhibits spectro-temporal transients which cannot be factorized into a product of a spectral and a temporal modulation. With this methodology, it is shown that approximately half of 52 analyzed zebra finch STRFs can each be well approximated by a single Gabor or a linear combination of two Gabor filters. Moreover, the dominant Gabor functions tend to be oriented either in the spectral or in the temporal direction, with truly “diagonal” Gabor functions rarely being necessary for reconstruction of an STRF's main characteristics. As a toy example for the applicability of STRF and Gabor-STRF filters to auditory detection tasks, we use STRF filters as features in an automatic event detection task and compare them to idealized Gabor filters and mel-frequency cepstral coefficients (MFCCs). STRFs classify a set of six everyday sounds with an accuracy similar to reference Gabor features (94% recognition rate). Spectro-temporal STRF and Gabor features outperform reference spectral MFCCs in quiet and in low noise conditions (down to 0 dB signal to noise ratio).

## Introduction

Robust detection and identification of behaviorally relevant sounds in possibly adverse acoustic conditions is routinely performed by animals and humans. In order to achieve this superior performance, the auditory system is thought to extract acoustic features from incoming sounds that are well-tuned to sound components facilitating acoustic event detection and discrimination (e.g., Lewicki, 2002; Coath and Denham, 2005; Smith and Lewicki, 2006). Applications such as computational auditory scene analysis, automatic speech recognition and signal enhancement may highly benefit from identifying and employing similar features. At the same time, performance of artificial systems equipped with nature-inspired acoustic feature extraction may provide a quantitative, albeit indirect, measure of those features in human and animal listening tasks.

While the auditory periphery is comparably well-characterized by experimental techniques, understanding processing in the inferior colliculus and auditory cortex requires a combination of experiment, data analysis and modeling. The spectro-temporal receptive field (STRF, Aertsen and Johannesma, 1981) represents a linear approximation to an auditory neuron's response characteristics. It is estimated from presented acoustic stimuli and recorded spike responses using the reverse correlation method or related statistical techniques (Bussgang, 1952; Aertsen and Johannesma, 1981; Chichilnisky, 2001). Since the obtained STRF pattern is the result of a combined stimulation, neuronal processing and statistical estimation procedure, it generally depends on a multitude of factors including animal species, stimulus ensemble, linear, non-linear, static and time-varying neuronal response characteristics, as well as the statistical inference method employed. STRFs have been measured in various species such as frogs (Eggermont et al., 1983; Bibikov, 1987), cats (Valentine and Eggermont, 2004), ferrets (Depireux et al., 2001), rats (Poon and Yu, 2000), gerbils (Lesica and Grothe, 2008), and birds (Theunissen et al., 2000; Woolley et al., 2005), showing that qualitative STRF characteristics are to a certain extent preserved across species and stimulus ensembles. Current ridge regression methods for performing reverse correlation obtain STRF estimates that are robust under variations of second-order correlations in the stimulus ensemble, including to some extent natural stimuli (Theunissen et al., 2000; Chichilnisky, 2001; Escabi and Schreiner, 2002; Paninski, 2003; Klein et al., 2006). Limitations to the linear and time-invariant STRF model have been investigated by several authors. They may result from higher-order statistics or non-stationarity in stimulus ensembles, non-linear neuronal processing or neuronal plasticity (Sahani and Linden, 2003; Kvale and Schreiner, 2004; Machens et al., 2004; Valentine and Eggermont, 2004; Fritz et al., 2005; Nagel and Doupe, 2006; Christianson et al., 2008) and require specific algorithms for the reliable estimation of underlying STRFs (Sharpee et al., 2004; Meyer et al., 2014a,b, 2015).

Typical STRFs display neuronal sensitivity that is localized in a short temporal window prior to spike generation and within a limited spectral range around the acoustic center frequency. Spectro-temporal sensitivity patterns often correspond to temporal or spectral onset processing; tuning to combined spectro-temporal transients has also been reported (cf. for example Versnel et al., 2009; Andoni and Pollak, 2011). Gabor basis functions have been proposed as a model for these observed two-dimensional spectro-temporal dynamics in STRFs, resulting in a family of functions that are parameterized in terms of acoustic center frequency, temporal position, spectral rate of modulation and temporal rate of modulation (Jones and Palmer, 1987; Qiu et al., 2003). A more general approach was pursued by Lindeberg and Friberg (2015), who presented a theoretical framework for spectro-temporal representations of sound, of which Gabor filters (among others) can be derived. They could show that their approach replicates STRFs found in a wide range of literature. Modulation analysis in spectral sub-bands (Kollmeier and Koch, 1994; Kingsbury et al., 1998; Jepsen et al., 2008) provides a model for processing of temporal dynamics only, e.g., using a bank of modulation bandpass filters or through delta and double-delta filters from automatic speech recognition (Moritz et al., 2015).

The goal of the present study is to quantitatively investigate spectro-temporal characteristics of physiologically measured STRFs. We propose a two-dimensional matching pursuit approach to approximate STRFs in terms of Gabor functions. Thus, Gabor functions serve as “atoms” during the matching pursuit estimation process that approximates STRFs with a sparse combination of Gabor patterns. Dominant spectro-temporal characteristics are retained during the estimation process. Minor variations are discarded depending on a reconstruction threshold that is varied as an independent parameter in our experiments, resulting in a compressed representation that resembles reduced redundancy in neural coding. Subsequent analysis of the parameters pertaining to those Gabor atoms that have been identified as dominant through matching pursuit, permits a quantitative characterization of the relevant spectro-temporal characteristics that are found most frequently in observed STRFs. These analyses implicy relative importance of purely spectral, purely temporal and joint spectro-temporal components in STRFs.

Applications from audio signal processing, computational auditory scene analysis and automatic speech recognition make use of modulation features in order to more accurately model natural signals (Lobo and Loizou, 2003; Turner and Sahani, 2007; McDermott and Simoncelli, 2011) and increase robustness under variability of environmental conditions such as recording channel, additive noise and speaker/target characteristics (Kleinschmidt and Gelbart, 2002; Mesgarani et al., 2006; Chu et al., 2009; Bach et al., 2011). Identification of those components in STRF patterns that represent salient features for automatic detection and recognition is thus important for development of robust recognition algorithms (Hermansky, 1998; Thomas et al., 2010). Compact feature sets comprising a small number of spectro-temporal basis functions are preferable from a numerical efficiency and statistical estimation perspective. Thus, investigation of the relation between STRFs and their dominant Gabor model components in the context of acoustic event detection may yield such a compact feature set for recognition algorithms. We explore the use of STRF-based feature extraction as front-end in an audio classification task, whose main aim is to identify strengths and weaknesses of the STRF approach in general, on a well-defined and manageable set of tasks, and to propose ways to improve on these in future work. Using classification accuracy as a performance measure, we compare STRF front-ends with Gabor front-ends and with Mel-frequency cepstral coefficients.

## Methods

### STRF estimation

The classic approach to STRF estimation is reverse correlation between stimulus and response (Bussgang, 1952; Aertsen and Johannesma, 1981; Chichilnisky, 2001). Let $\text{s}={\left(s_{1}s_{2}\ldots s_{D}\right)}^{T}$ denote the spectro-temporal stimulus pattern preceding the response in a specific time window, recast as a *D*-dimensional vector. *r* is the corresponding response value estimated from multiple stimulus repetitions. Without loss of generality, we assumed that the stimulus vectors have a mean of zero. The reverse correlation function can be estimated as the spike-triggered average:

(1) $${\text{h}}_{\text{RC}}\propto {\langle \text{s}r\rangle }_{s},$$

where 〈·〉_*s*_ denotes expectation over the whole stimulus ensemble. **h**_RC_ is a vector with the same size as the spectro-temporal stimulus patterns used for the estimation, and indicates the stimulus features to which the neuron is most sensitive.

Natural signals are often correlated across time and frequency and have on several occasions been reported to exhibit a 1/*f*-like power spectrum. (Voss and Clarke, 1975; Attias and Schreiner, 1997; Woolley et al., 2005). Correlations can be removed using the (pseudo-)inverse of the stimulus auto-covariance matrix ${\langle \text{s}{\text{s}}^{T}\rangle }_{s}$ (Theunissen et al., 2000). To avoid overfitting, we used a regularization scheme based on ridge regression (Machens et al., 2004):

(2) $${\text{h}}_{\text{ridge}}\propto {\langle \text{s}{\text{s}}^{\text{T}}+\lambda \text{I}\rangle }^{-1}\langle \text{s}r\rangle .$$

where **I** is the identity matrix and λ ≥ 0 is the regularization parameter, ranging from a non-regularized solution (λ = 0) to the original reverse correlation function (Equation 1) for λ → ∞. We used this estimate as a regularized, whitened, spike-triggered average (STA).

The STRF estimation based on STA was repeated using a bootstrap procedure: 20% of the spike data was drawn randomly and an STRF was estimated based on those items. This was repeated 1000 times, and the mean and standard deviation of each STRF coefficient over the 1000 repetitions were computed. Only coefficients that significantly (*p* < 0.05) differed from the null hypothesis were kept, the rest was set to 0, resulting in visually “clean” STRFs (Escabi and Schreiner, 2002). Non-zero spectro-temporal “pixels” of size 1x1 that were completely surrounded by zero components (i.e., considering the 3 × 3 spectro-temporal patch centered around the non-zero pixel) were regarded as insular artifacts and removed.

The correlation between neural response and model prediction was computed depending on the regularization parameter. In a 5-fold cross-validation setting, we used the regularization parameter that resulted in the highest mean correlation (mean over cross-validation iterations) for STRF estimation. Furthermore, we only included STRFs that yield a mean correlation of at least 0.25 on previously unseen data, resulting in a total of 52 STRFs.

While experimental sampling of auditory areas is generally sparse relative to the total number of auditory neurons, the shapes of the measured receptive fields were assumed to be representative of the whole neuronal population. Thus, cells with similar spectro-temporal sensitivities were also expected to exist for different best frequencies. While experimental limitations do not allow a rigorous proof of this assumption, it plausibly lends itself to the approach of STRF pattern replication across frequency as pursued here (see below).

### Gabor filter bank

Gabor functions constitute a quantitative description of two-dimensional spectro-temporal filters for feature extraction. They have been widely used in image processing, but successful applications are also found in audio event detection (Chu et al., 2009) and automatic speech recognition (Meyer et al., 2011). Gabor filters have been put forward as models for receptive fields in vision as well as audition. They lend themselves naturally to two-dimensional pattern recognition in terms of modulations, with Gabors of different widths covering different transfer properties. Gabors are constant-Q filters when the number of oscillations under the envelope is held fixed, similar to (Morlet) wavelet analysis. Here, Gabor filters were defined as a complex sinusoidal carrier with a Hann envelope:

(3) $$\begin{array}{l} g(t,f)=s(t,f)h(t,f)\\ \;=s_{\omega_{t}}(t)s_{\omega_{s}}(f)h_{b_{t}}(t)h_{b_{s}}(f) \end{array}$$

where

(4) $$s_{\omega }(x)=e^{i\omega x}$$

(5) $$h_{b}(x)\;=\{\begin{array}{ll} 0.5-0.5\cos\left(\frac{2\pi x}{b}\right) & ,-\frac{b}{2}<x<\frac{b}{2}\\ 0 & ,\text{else} \end{array}$$

**Figure 1:**
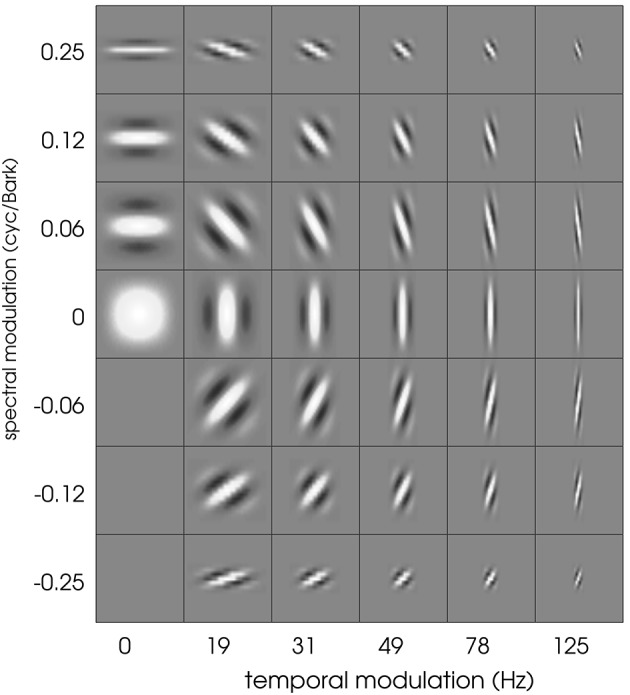
**The full Gabor filter bank**. Each square has a width of 100 ms and a height of 21 Bark channels.

Schädler et al. (2012) designed a filter bank of Gabor filters whose transfer functions uniformly cover the temporal and spectral modulation subspace (spanned by the spectral and temporal resolution of the 2D representation). We used the same filter bank design, adapting the parameters to the employed spectro-temporal peripheral model. The spectral extent of the Gabors was limited to 21 bands (size of the STRFs). The cited procedure lead to Gabor filters covering spectral modulations between 0 and 0.25 cyc/Bark and temporal modulations between 0 and 125 Hz. The real parts of the resulting (complex-valued) Gabor filters are shown in Figure 1.

### Spectro-temporal convolution with STRF and gabor functions

**Figure 2:**
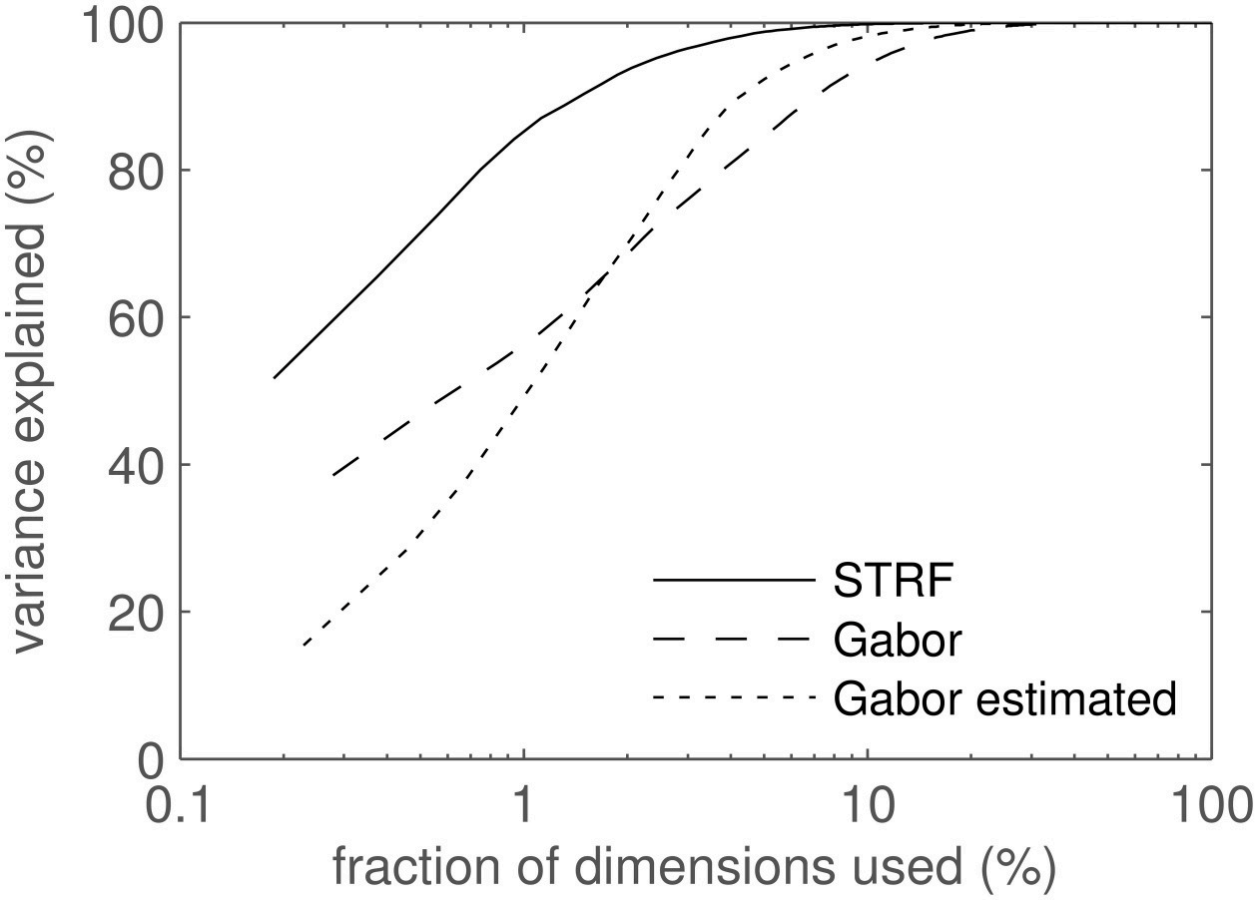
**PCA results**. Cumulative variance explained by the PCA components plotted against the number of feature dimensions used. Since STRF, Gabor, and estimated Gabor feature spaces have different dimensionalities, values along the abscissa are relative. The abscissa is log-scaled to better visualize the interesting region of low dimensionality.

STRF- and Gabor-derived features for classification were computed by 2D-filtering of spectrograms. Using all 52 STRF patterns as 2D filters resulted in 1092 feature dimensions (21 frequency channels × 52 filters). The number of feature components could be reduced by exploiting the high correlation found between the outputs of adjacent frequency channels in STRFs with large spectral extent. Since highly correlated feature components can result in reduced classifier performance, a number of representative channels was selected by sub-sampling the 21-dimensional output of each STRF-based filter. For Gabor filters, the filter width was computed as the 1/*e* decay point of the envelope; for STRFs, the filter width was estimated as the spectral width containing 90% of the energy of the filter. Channels closer than $\frac{1}{4}$ of the filter width in spectral direction are dropped, cf. Schädler et al. (2012) for details. Finally, Principal Component Analysis (PCA) was performed to decorrelate the feature dimensions. Figure 2 displays the amount of variance in the data cumulatively explained by the PCA components. STRF features have the steepest PCA curves, i.e., their variance can be explained with the lowest number of dimensions. This arises from the strong similarity between some STRFs which causes them to produce highly correlated features. By construction, the Gabor filter set consists of highly non-redundant filters. The filter shift along frequency (see above) as well as some spectro-temporal overlap between the filters causes some feature channels to be (weakly) correlated. Table 1 shows the number of dimensions needed to account for 90 and 99% of the data. The high numbers for Gabors and the low numbers for STRFs illustrates the high degree of redundancy in STRFs compared to Gabor filters.

**Table 1:** **PCA results**.

**Variance Explained**	**Feature set**	**Dimensions**
90%	STRF	8 (1%)
90%	Gabor	26 (7%)
99%	STRF	30 (6%)
99%	Gabor	74 (21%)

*Results of the principle component analysis: number of features needed to account for 90 and 99% of the variance. The second column contains the feature sets. “STRF”: estimated neural responses; “Gabor”: Gabor filter bank (Figure 1). The third column gives the number of dimensions; percentages relative to the dimensionality of full feature set are given in parentheses*.

In the experiments section, some results are given in terms of accuracy against relative amount of variance explained by the PCA in order to compare the different feature sets. We use variance as an (arguably imperfect) estimator of information content, assuming Gaussian distributions throughout.

### Matching pursuit analysis of STRFs with gabor atoms

As described above, Gabor filters have been proposed as models for STRFs. Explicitly describing the STRFs found in the present work in terms of the Gabor filter bank underscores this choice. This was done by implementing a two-dimensional matching pursuit (MP) algorithm (Mallat and Zhang, 1993). MP is a greedy reconstruction algorithm of signals by a dictionary of given “atoms.” In our case, the target “signals” were the STRFs, and the atoms were defined by the elements of the Gabor filter bank. MP computes the overlap γ between signal and each atom in the dictionary by correlation. At each iteration step *i*, γ_*ijk*_ was defined as the maximal correlation coefficient, computed from two-dimensional correlation between the *j*-th Gabor atom and the *k*-th STRF. Let *CC* denote two-dimensional cross-correlation, *g* the Gabor function as defined above, and *S* the STRF:

(6) $$\gamma_{ijk}={\max\{CC(g_{j},S_{k})\}|}_{\text{iter}=i}$$

γ was computed for all Gabor atoms, the projection of the atom with the largest correlation coefficient was subtracted from the signal, and the process was repeated on the residual signal. The correlation coefficient θ between original and reconstructed STRFs increases monotonically through MP iterations. For most tasks, we chose θ = 0.8 as termination criterion. As described above, the Gabors are complex-valued filters; for MP, we used the real (symmetric) and imaginary (antisymmetric) parts as independent, real-valued Gabor atoms. Re-synthesized STRFs were in turn used for feature extraction and subsequent classification. The effect of the MP re-synthesis on these features was two-fold: first, the filter shapes are approximated by smooth Gabor shapes, i.e., irrelevant noise and artifacts in the STRF patterns may be removed when the reconstruction is sufficiently coarse. Second, from an information point of view, approximating filters incompletely results in a loss of information.

**Figure 3:**
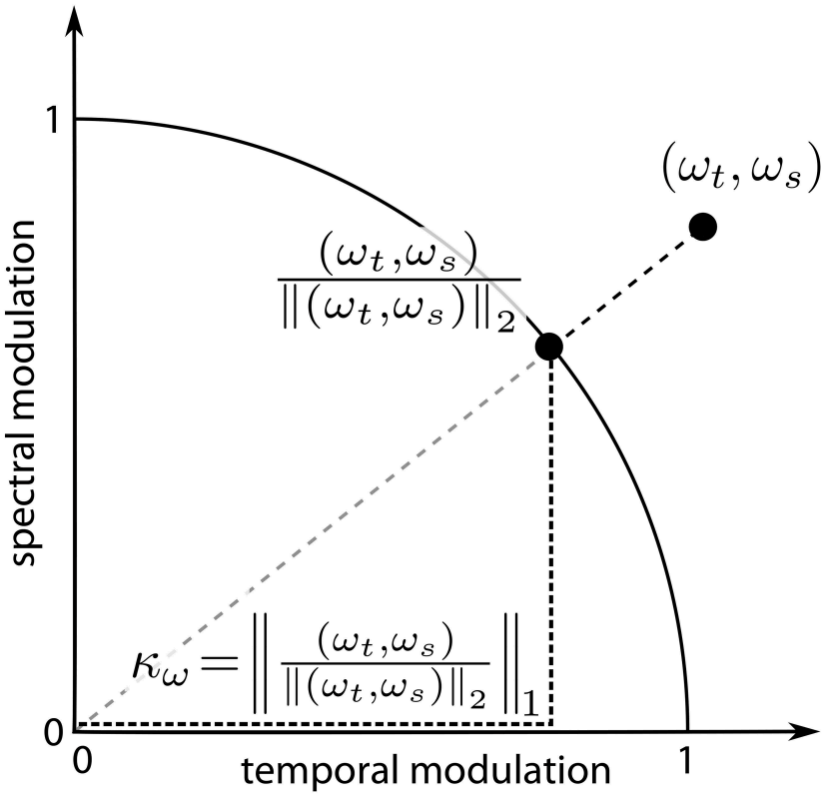
**Computation of the measure of spectro-temporality κ_ω_**. The modulation vector **ω** = (ω_*t*_, ω_*s*_) is normalized to unit length; κ_**ω**_ equals the L1-norm of the normalized vector.

For further analysis, we defined a measure κ of “spectro-temporality” of the reconstructed filters, which is computed as follows: the vector of modulation frequencies of a Gabor atom, **ω**, was L2-normalized, i.e., it was projected to the unit circle in modulation space. κ was then computed as its L1-norm (cf. Figure 3). Let **ω** = (ω_*t*_, ω_*s*_) be the vector of temporal and spectral modulation frequency of a Gabor atom. For each element of **ω**: π/2 ≤ ω_*t*|*s*_ ≤ π/2. Then the spectro-temporality measure κ_**ω**_ of this Gabor atom is defined as the L1-norm of the L2-normalized frequency vector:

(7) $$\kappa_{\omega }={\left\|\frac{\omega }{{\left\|\omega \right\|}_{2}}\right\|}_{1}=\frac{\left|\omega_{t}|+|\omega_{s}\right|}{\sqrt{\omega_{t}{}^{2}+\omega_{s}{}^{2}}}$$

κ ranges from 1 (purely spectral or purely temporal) to $\sqrt{2}$ (diagonal). In the MP task, the value κ assigned to the MP reconstruction of an STRF over *N* iterations was the average of the κs of the Gabor atoms involved:

(8) $$\kappa =\frac{1}{N_{\text{iter}}}\sum_{i=1}^{N_{\text{iter}}}{\left(\kappa_{\omega }\right)}_{i}$$

### Neural processing model for acoustic event detection

**Figure 4:**
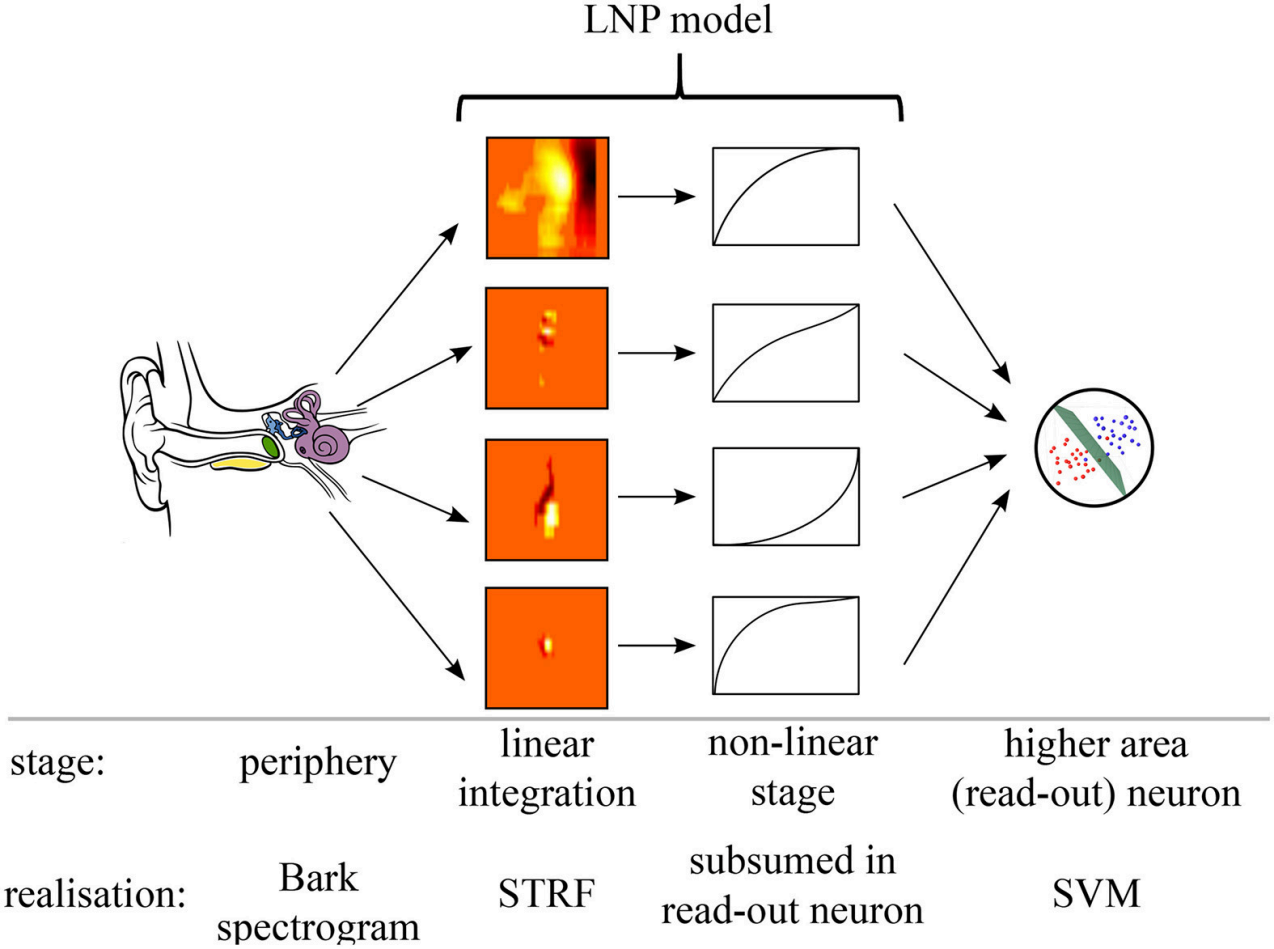
**Processing model**. Illustration of the processing stages in the assumed sound detection architecture, comprising auditory periphery, LNP model, and detection neurons.

Figure 4 illustrates the linear-non-linear Poisson model (LNP) of neural processing that was employed for sound-event detection in the present work. Relevant functional processing steps included cochlear transformation of incoming sound into the corresponding time-frequency representation, extraction of spectro-temporal features in a bank of parallel simple receptor neurons with linear STRFs, a non-linear response stage, and integration of neuronal activities into a single event detection output through one downstream read-out neuron per acoustic event class. STRFs in the feature extraction layer are derived from (recorded and simulated) neuronal responses to acoustic stimuli and remain fixed after their initial estimation. The subsequent read-out neuron was trained using supervised machine learning methods on examples of acoustic event data. This architecture resembles a classical hierarchical feed-forward processing approach, with feed-forward connectivity estimates derived from physiological data, combined with a non-linear classifier architecture in the read-out stage. Previous studies analyzed, in a feature-driven approach similar to the one pursued here, how physiologically motivated sound processing performs in recognition and sound segregation tasks by using multi-dimensional mappings, see for example Elhilali and Shamma (2008). Algorithms from computer vision were adopted in auditory models of peripheral and higher processing (Lyon et al., 2010).

## Experiments and results

### Overview

Results are organized in two parts. In part (A), we analyze the spectro-temporal properties of STRF patterns resulting from the STA estimation process both quantitatively and qualitatively: From filter shapes, we deduce typical properties of STRFs. Using a matching pursuit approach, we re-synthesize STRFs from Gabor filter atoms. Analysis of the Gabor atoms used in the process results in a quantitative characterization of spectral, temporal, and spectro-temporal properties of the STRFs. In part (B), we use a data set of acoustic event recordings as a toy example for application of STRF-based and Gabor filters as a front-end for sound classification. This experiment investigates how the STRF-based features perform compared to Gabor filter-based features and to (purely spectral) Mel-frequency cepstral coefficient (MFCC) features in quiet and in noisy conditions.

### A: STRF estimation and analysis of spectro-temporal properties

Neural data from several areas in the zebra finch (*Taeniopygia guttata*) auditory system was used to compute auditory STRFs, cf. Gill et al. (2006) for a detailed description of the experimental setting and data. Single-unit recordings were taken from anesthetized zebra finches. The animals listened to stimuli consisting of conspecific songs and modulated ripple patterns that had the same modulation spectrum as conspecific bird song. Recordings were performed in areas Caudal Mesopallium, Primary Forebrain (from sub-areas L1, L2a, L2b, L3, and L), Nucleus Ovoidalis, and Mesencephalicus lateral dorsalis. Stimuli were typically repeated 10 times.

**Figure 5:**
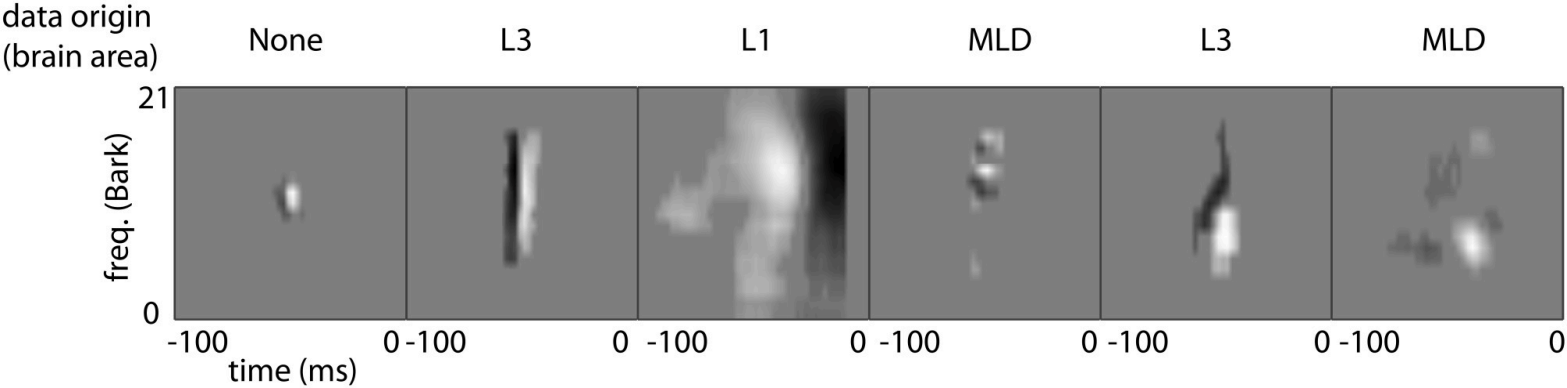
**Typical shapes of STRFs found in this study**. The two examples on the left depict STRFs corresponding to temporal onset detectors of varying spectral bandwidths. The third panel has a similar detector characteristics for broadband offsets. The fourth pattern represents a detector for spectral modulations, while the 5th and 6th panels show more complex transient spectro-temporal behavior.

Typical shapes of auditory STRFs are displayed in Figure 5. These correspond to narrowband and broadband onset/offset detectors, frequency modulation detectors, transient detectors, and complex spectro-temporal patterns. Most STRFs observed can be classified as one of the first three groups.

**Figure 6:**
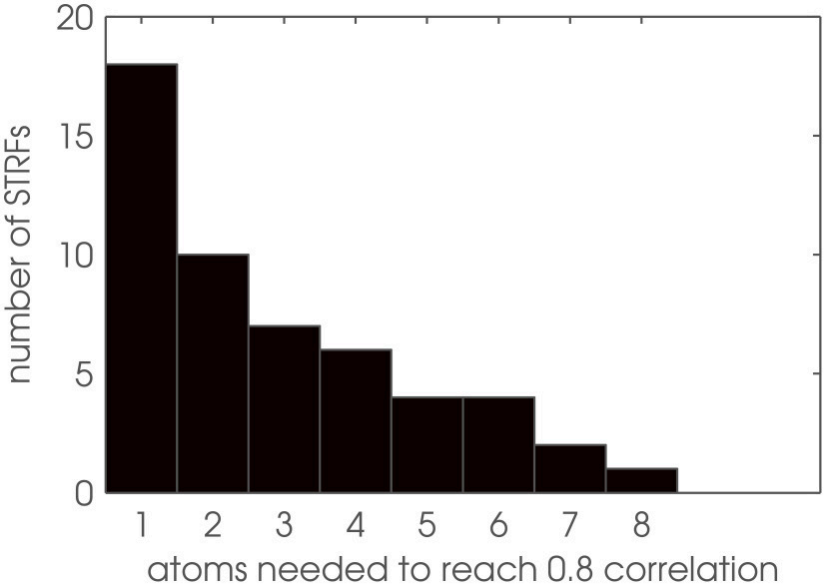
**Dimensionality analysis of STRF patterns**. Number of Gabor atoms needed to reconstruct STRFs with θ = 0.8 in matching pursuit.

Based on these findings, we used Gabor filters as model patterns for auditory STRFs by approximating the zebra finch STRFs with Gabor basis vectors using two-dimensional matching pursuit (MP). In a first step, we determine the number of Gabor atoms needed to reproduce an STRF pattern with sufficient reconstruction accuracy. Figure 6 shows a histogram of the results: More than half of the 52 STRFs need only 1 or 2 Gabor atoms. The single most complex pattern is represented by a superposition of 8 Gabor atoms. Thus, STRFs can be well approximated as sparse combinations of Gabor basis functions, with a comparably low dimensionality of each STRF pattern when decomposed into Gabors.

**Figure 7:**
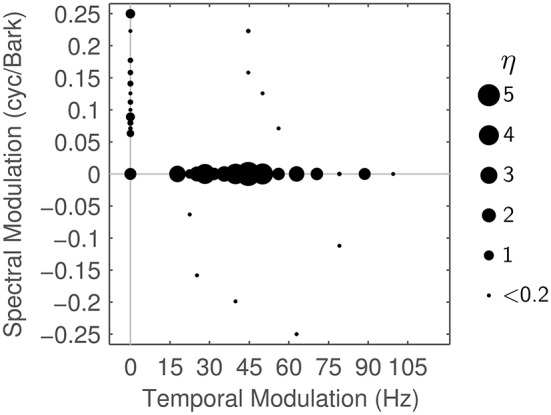
**Matching Pursuit: Modulation characteristics**. Importance η of the different Gabor atoms when using matching pursuit (θ = 0.8), plotted by modulation frequencies (see text for details).

In a second step, we analyze the particular Gabor shapes that are used in reconstructing the STRF in terms of their spectral, temporal and joint spectro-temporal extent. Figure 7 shows the dominant modulation frequencies contained in the reconstructed STRFs. These are discretely spaced because each Gabor atom corresponds to one specific spectro-temporal modulation. The area of each disc in the figure is proportional to the importance η_*j*_ of the *j*-th Gabor atom in the MP task. η_*j*_ is computed as the overall weight from the γ_*ijk*_ (cf. Equation 6) the atom received in all iterations of MP for all STRFs:

(9) $$\eta_{j}=\sum_{i=1}^{N_{\text{iter}}}\sum_{k=1}^{N_{\text{STRF}}}\left|\gamma_{ijk}\right|$$

**Figure 8:**
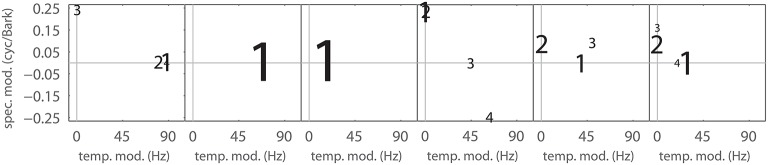
**Matching Pursuit: Gabor weight analysis**. First up to 4 Gabor components needed to approximate the filters shown in Figure 5 with an MP threshold of θ = 0.8. Font size corresponds to the MP weight γ of the Gabor atom. The panels left to right correspond to the panels in Figure 5.

Figure 7 shows η_*j*_ for all Gabors, computed at a reconstruction threshold of θ = 0.8. In combination with the modulation frequencies of the Gabor atoms used in the first couple of MP iterations for typical STRFs (Figure 8), the surprising result is that even with transient STRF patterns, i.e., STRFs with simultaneous spectral and temporal modulations, virtually no Gabor atoms with diagonal shape are used in MP. This could be a result of the chosen threshold value (θ = 0.8) under the hypothesis that the more detailed the reconstruction (higher θ), the more likely the use of diagonal atoms is. The threshold θ = 0.8 might be comparatively low, as may be indicated by the low number of Gabors needed (Figure 6).

**Figure 9:**
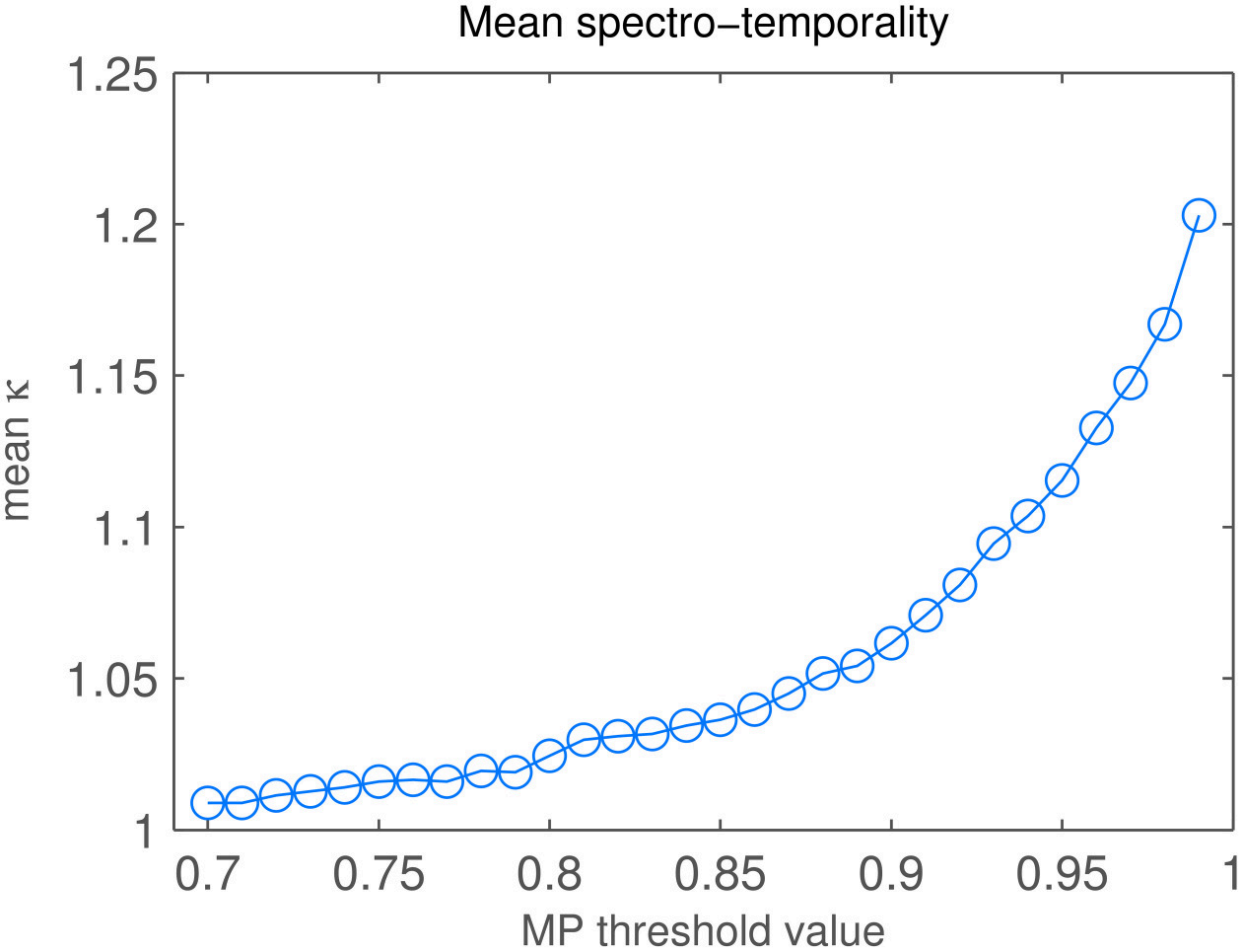
**Matching Pursuit: Spectro-temporality analysis**. Diagonality κ of STRFs, depending on MP threshold value.

Varying the reconstruction threshold in the MP task changes the number of iterations and hence changes the spectro-temporality index κ (Equation 8). Figure 9 shows the change of κ when varying the MP threshold between 0.7 and 0.99. Up to about θ = 0.8, κ shows a slow rise but stays below 1.02. From θ = 0.81 to 0.99, we find a rise in κ with increasing slope, i.e., a higher number of diagonal atoms is needed to reconstruct the fine details of the STRF patterns.

### B: Application in sound classification

#### Acoustic event data

To assess the use of STRFs for automatic event classification, we recorded a corpus of acoustic events and evaluated accuracy of the neural processing model (cf. ) in a 6-class classification task. The 6 classes were distinct everyday sound objects: speech, a ringing telephone, a running tap, an electrical toothbrush, a coffee grinder, and clinking glasses. Several hundred events (approximately 10 min of data) per class were used for training, and about half as much (5 min) for testing; all data were recorded in-house except speech, which was taken from the TIMIT database (Garofolo et al., 1993). For the in-house recordings, we used different rooms and settings for training and testing: glass clinks, water tap, coffee grinder and telephone sounds were recorded from at least two different types of devices each. Recordings of telephone and coffee grinder had natural beginning/end points since they operated in an automated way (a couple of seconds per run). Each device was recorded in all of its operation modes (telephones: 8 different ring tones each, coffee grinders: 6 different settings each) at least 100 times. The glass clinks were recorded continuously by clinking a number of glasses repeatedly against one another without silent intervals in between. This was continued for about 2 min and repeated 10 times. The same procedure was repeated with different sets of tumblers and glasses in a different room for recording the test set. Segments were arbitrarily cut out of the continuous clinking. Experiments in noise were done using artificial noises (stationary Gaussian ICRA-1, speech simulating multi-speaker babble ICRA-7, Dreschler et al., 1999) as well as recorded noise (pedestrian zone noise and road noise, Bach et al., 2011). The noises were chosen to cover the spectral and modulation ranges of the sound classes.

The peripheral model employed in the detection model is a Bark-scaled spectrogram representation. We used 4 ms windows with 2 ms overlap to compute the linear spectrogram (128 point discrete Fourier transform) at a sampling rate of 16 kHz. This was followed by a trapezoid Bark summation with a bandwidth of 1 Bark. Amplitudes are compressed by cubic root compression. The resulting spectrogram had 21 Bark channels and a temporal resolution of 2 ms.

Extraction of Mel-frequency cepstral coefficients (MFCC, Davis and Mermelstein, 1980) provided baseline features that served as a comparison to STRF and Gabor features. The reference implementation used here (Ellis, 2005) is compatible with the HTK standard, which uses a 25 ms frame length, 10 ms window shift, 23 triangular Mel filters, and logarithmic amplitude compression. The first 13 coefficients were used, the first one replaced by the log-energy of the signal. Extracting the derivative and acceleration parameters (Δ and ΔΔ features) for all 13 dimensions using 7-point and 9-point linear slopes resulted in a 39-dimensional feature vector per 10 ms time step.

The classification experiments used Support Vector Machines (SVM) as discriminative models. We trained 1-vs.-all models and perform classification with a winner-take-all strategy. Since we used radial basis function (RBF) kernels, the SVM penalty parameter and the radius of the basis functions had to be fixed. SVM parameter optimization was conducted by grid search with 5-fold cross validation to find the optimal values.

We analyzed the performance of STRF-based 2D filters in the classification task with respect to four different effects: temporal resolution of the spectral representation and comparison between spectral and spectro-temporal features, effect of the MP reconstruction, and robustness against noise.

**Figure 10:**
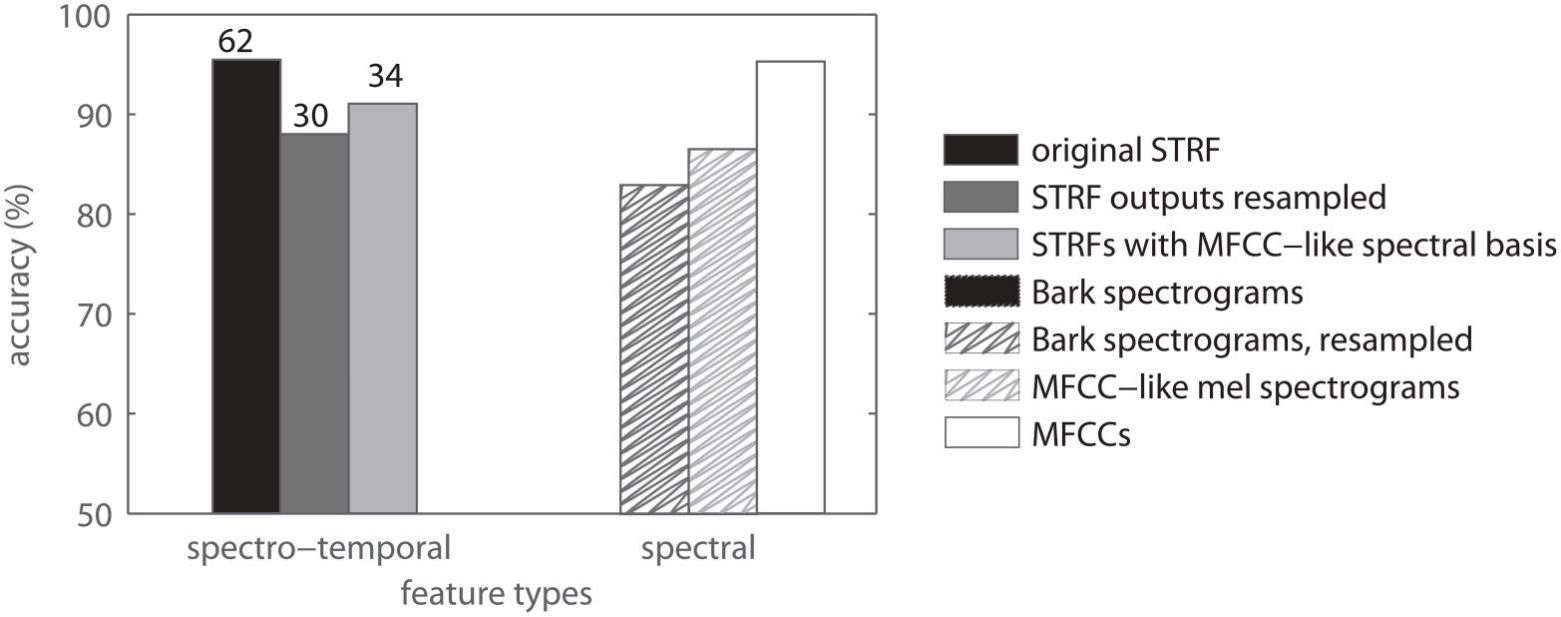
**Comparison of spectral and spectrotemporal patterns for classification**. The left set of bars refers to spectro-temporal features, the right set of bars refers to spectral features. Numbers on top of the spectro-temporal data indicate relative reduction of errors in %. Note that these data were obtained with feature sets of comparable dimensionality.

The spectro-temporal filters analyzed in the above experiments share a common peripheral pre-processing. The spectral and temporal parameters of the peripheral processing are made to fit the neural data in order to produce meaningful STRF shapes. In particular, a comparatively high temporal resolution (≈ 2 *ms*) is needed to estimate meaningful STRFs from avian auditory data. The audio event detection literature suggests that parameter settings similar to those typically used in speech processing are better suited to general acoustic events, too (Cai et al., 2006; Aucouturier et al., 2007; Bach et al., 2011). We therefore presume that the parametrization may be a limiting factor for the later processing stages. We allow for that effect by comparing the performance of features derived with different spectral parameter settings. In particular, we used the following variants of STRF-based features: (a) resampling of the STRF filter outputs to the temporal resolution of the MFCC baseline. (b) resampling of the original STRFs to the lower temporal resolution and using them as 2D filters on spectrograms with the same low resolution (i.e., spectrograms comparable to those used to compute MFCCs). These different spectro-temporal features are compared to the same features with no STRF processing, i.e., different sets of spectrogram features, and with MFCCs. Figure 10 shows the results. The left set of bars shows results of spectro-temporal features while the right set of bars shows results of spectral features. The difference between left and right bars of the same color, shown as reduction of errors (in %), is the relative “spectro-temporal benefit” resulting from using STRF-type filters. This benefit is largest when using high resolution STRFs (black bars) and smallest when using a spectrogram basis as used for MFCCs. (light gray bars).

**Figure 11:**
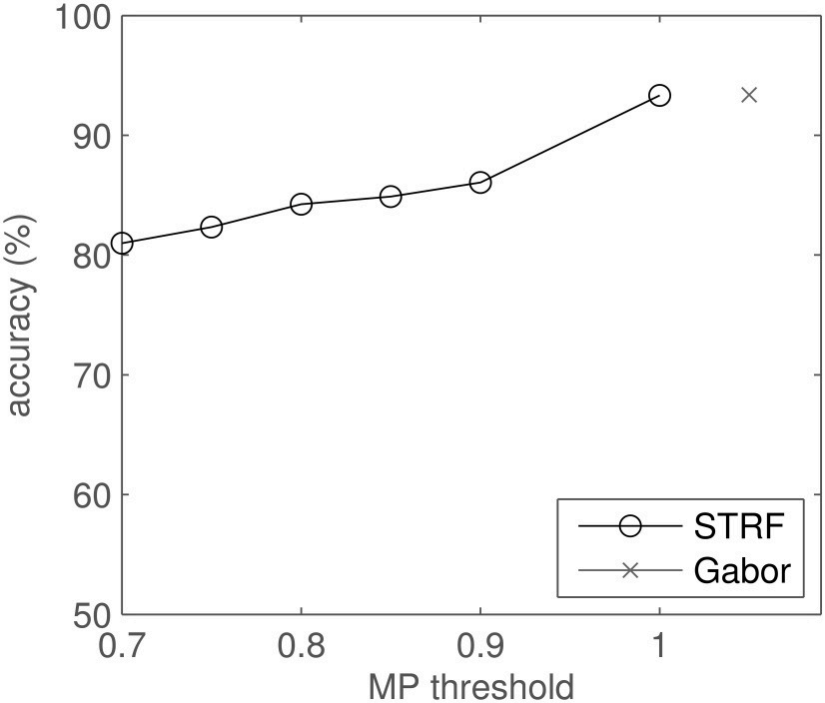
**Classification results for MP-reconstructed STRFs using several MP thresholds and comparison to Gabor filter-based results**. Note that the abscissa does not apply to the Gabor result.

The effect of the MP reconstruction on the classification results is shown in Figure 11. The plot shows classification accuracy as a function of reconstruction threshold θ. Each approximation, i.e., each drop in θ, results in a (small) drop in performance.

**Figure 12:**
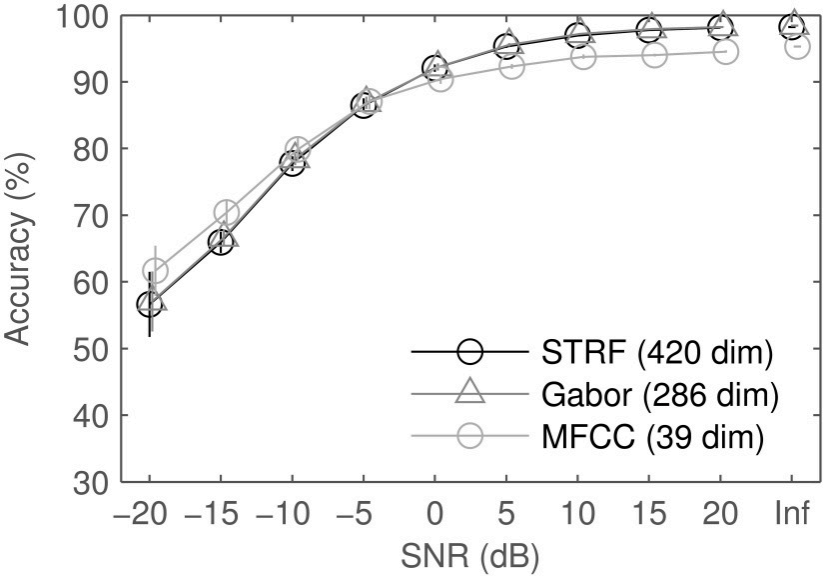
**Results of the different feature sets in noise: average and standard error of the mean over 4 different noises**. SNRs were identical for all feature sets, shifts on the abscissa are for visual clarity only. For comparison, results on clean data are also displayed as SNR: Inf.

We tested the robustness against noise by mixing the audio data in four different background sounds, one stationary (and artificial), three modulated (one of them artificial). Figure 12 shows the mean results and standard error of the mean, computed over the four noise conditions. Spectro-temporal features perform better in high and moderate SNRs. At low SNRs, MFCCs beat both STRFs and Gabors. Standard errors computed over noises are much larger at low SNRs, indicating that performance varies depending on the type of noise. Detailed results for each noise type (not shown) reveal that the general trend is similar for all noise types, we therefore omit more detailed plots. In real recorded noise (road noise, pedestrian zone noise), spectro-temporal patterns are slightly closer to par with MFCCs than in both modulated and unmodulated artificial noise (ICRA-1, ICRA-7).

## Discussion

The STRFs analyzed in this work were found to be spectro-temporal patterns with largely separable spectral and temporal properties. We did not pursue a specific analysis of separability. Previous studies have shown explicitly that auditory STRFs in mammals are to a large extent separable. See e.g., Depireux et al. (2001) and Versnel et al. (2009) for corresponding studies in ferrets and macaques, respectively.

**Figure 13:**
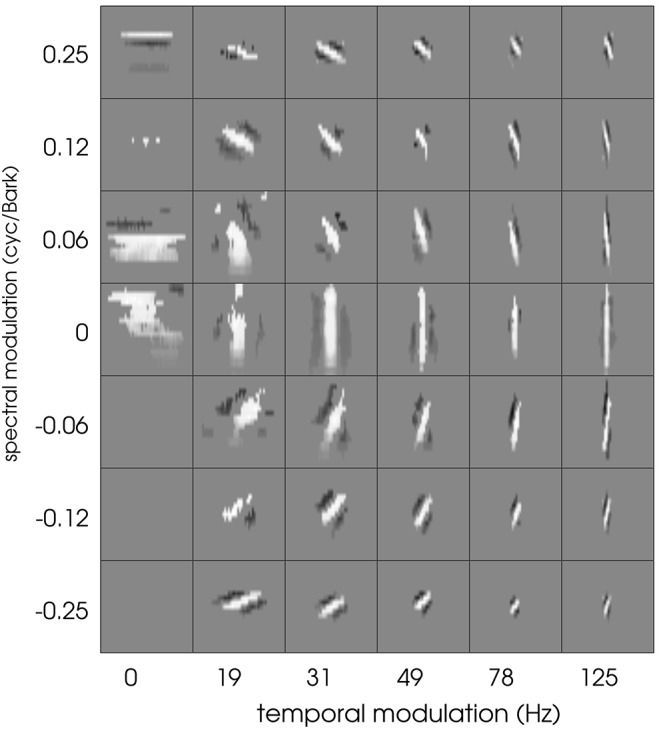
**The full Gabor filter bank after re-estimation using the LNP model and STA estimation**. See text for details. Each square has a width of 100 ms and a height of 21 Bark channels. Cf. Figure 1 for comparison.

Typical patterns found in the current data set include (a) temporal “onset detectors” with different spectral bandwidth and differing temporal extent, (b) spectral modulation detectors with virtually no variation in temporal direction, and (c) transient and more complex patterns. Results found in matching pursuit experiments show that each of these filters was adequately represented by a small number of well-designed Gabor filters. The question arises to what extent spectro-temporal properties are influenced by the limitation of the neural recordings and the linear estimation process. To this end, we employed the Gabor filter bank as a set of artificial STRFs. Non-linearities and a Poisson process were added after the linear stage to extend the Gabors to full LNP neurons. Using the same stimuli as employed in the original experiments (Gill et al., 2006), an electro-physiological experiment with resulting spike trains was thus simulated. STA computation based on these simulated spike trains was used to re-estimate the STRF of the artificial Gabor-based LNP neurons. Figure 13 shows the STA-estimated Gabor filter bank. STA obviously keeps spectro-temporal properties of the filters intact. However, we observe a loss of information for most of the purely spectral filters (first column of filters in Figure 13). For purely temporal filters (middle row), this detrimental effect is much less pronounced. In general, the STA process produces slightly washed-out versions of the Gabor filters.

Using Gabor approximations of STRFs also proved to be beneficial in the sense of efficiency in classification: at equal accuracies, these smoothed filters needed less relative information than the original STRFs. However, the original STRF filter bank still reaches higher scores (Figure 11, MP threshold = 1). This indicates that the information loss due to incomplete approximation by Matching Pursuit is the dominant effect compared to the hypothesized artifact-removal property of the process. This may possibly be explained by the fact that significance analysis and subsequent artifact removal were already implemented in the optimized STA estimation of STRFs, hence the MP yields no extra benefit. STRFs reach performance competitive to an idealized Gabor filter bank, both in quiet and in noise (Figures 11, 12). This indicates that the STRFs used in this work extract most of the relevant information, even given the limiting boundary conditions (spectral representation, temporal resolution).

A different approach was pursued by Lindeberg and Friberg (2015). Providing a theoretical framework for deriving spectro-temporal representations of sound, they could qualitatively reproduce a large number of STRF shapes found in a variety of animal studies. The strength of the theory lies in the large variability of the representations, encompassing (among others) common Gabor and Gammatone filter representations. However, due to the generic nature of the theory, their approach does not lend itself naturally to quantitative recovery with matching pursuit. Moreover, in this work we emphasize the simplification brought about by using a small number of Gabor shapes, with only two free parameters (namely temporal and spectral modulation). It may be interesting to pursue, in a data-driven approach, a variety of features provided by their approach, and find the best filter shapes for specific tasks (such as event detection, automatic speech recognition, and others). This is, however, beyond the scope of the current paper.

Pre-processing provides further room for improvement. The peripheral model is adapted from well-known human and animal perception models, which include amplitude compression and log-spaced frequency-specific filters with increasing bandwidth. The approach was *ad-hoc* adapted to fit the zebra finch neural data in order to obtain suitable STRFs. The temporal resolution of the spectro-temporal representation is clearly much higher than in speech processing, for example. We expect some improvement if the periphery was tuned toward the application rather than toward physiology. This approach was pursued in previous work by Mesgarani et al. (2006), replacing real STRFs with properly selected, application-oriented filters in a sound discrimination task. If one were to stick more closely to physiological data, different animal models may provide better data.

Ferrets, for example, have been shown to exhibit broader temporal patterns in their STRFs (Klein et al., 2006; Mesgarani et al., 2008). However, for the purpose of the current work, the set-up, size, and availability of the zebra finch data base was the best choice. Our results indicate that STRFs can provide large benefits in terms of relative reduction of errors. This overcompensates the less than optimal peripheral processing chosen here. Factoring in the aforementioned observation that the STRFs are mostly temporal or spectral, one is tempted to assume that the effect of STRF filtering is little more than temporal averaging, i.e., a simple low-pass filter, which in itself is potentially beneficial for classification since it removes high-frequency noise. Comparing the results of temporally smoothed spectrogram features with those of STRF features (Figure 10), however, it becomes obvious that the effect of STRFs is far stronger than that of temporal averaging.

## Conclusions

The experimental evidence presented here leads to several conclusions.

- Gabor filters are an appropriate model for auditory STRFs. The majority of STRFs is adequately modeled by at most two Gabor functions. No STRFs were more complex than the weighted sum of eight Gabors.
- Virtually all STRFs could be represented by a sum of purely temporal and purely spectral Gabor shapes, i.e., they contain separable spectral and temporal information. This is in line with findings from, e.g., Qiu et al. (2003). Similar results have been found for Gabor filters in automatic speech recognition (Schädler and Kollmeier, 2015).
- STRFs approximated with a sum of Gabor shapes are more efficient (in terms of dimensionality) in classification tasks than the original STRFs, albeit not reaching higher absolute scores. We conclude that the very compact STRF-based representation makes them a logical physiological model for feature extraction.

These results open up several avenues for future work. Different methods for STRF estimation may provide cleaner, more compact estimates. Examples include reverse correlation-based approaches with additional processing steps similar to the one pursued here, machine-learning- (Meyer et al., 2014a) or information theory-based approaches (Sharpee et al., 2004). Filters derived from such estimates may reduce redundancies in the features and span a larger effective feature space. Alternatives to single cell recordings might alleviate the restrictions set by the sparse sampling of brain areas. Electro-corticography, for example, allows similar reconstruction of spectro-temporal filters (based on reverse correlation) by measuring cortical response fields (Pasley et al., 2012); they provide the additional advantage that responses can be measured in human subjects (Mesgarani and Chang, 2012). Additionally, single cell neural data from different species allows comparison of different sets of filters. On the one hand, this may generally produce STRFs in different spectro-temporal parameter regimes, on the other hand, data from mammals for example may produce STRFs that are plausible for modeling human perception, including speech coding (Mesgarani et al., 2008) and phoneme classification (Herff et al., 2015).

## Author contributions

JB and JA designed the study, BK participated in designing the study. JB, BK, and JA participated in discussions. JB and JA devised methods and experiments and conducted analysis of results. JB implemented methods. JB and JA wrote the manuscript.

### Conflict of interest statement

The authors declare that the research was conducted in the absence of any commercial or financial relationships that could be construed as a potential conflict of interest.
